# Learning transfer from classroom to clinical practice in surgical units: a descriptive phenomenological study of undergraduate nursing students

**DOI:** 10.1186/s12912-026-04990-5

**Published:** 2026-07-07

**Authors:** Sevim Akbal, Havva Bozdemir, Meltem Yildirim

**Affiliations:** 1 Nursing Department, Kesan Health School, Trakya University, Edirne, 22030 Turkey; 2 Nursing Department, Health Science Faculty, Kocaeli University, Kocaeli, 4100 Turkey; 3 Research Group on Methodology, Methods, Models and Outcomes of Health and Social Sciences (M3O), Faculty of Health Sciences and Welfare, Centre for Health and Social Care Research (CESS), University of Vic-Central University of Catalonia (UVic-UCC), Vic, Spain; 4 Institute for Research and Innovation in Life Sciences and Health in Central Catalonia (IRIS-CC), Vic, Spain

**Keywords:** Undergraduate nursing student, Nursing education, Qualitative research, Clinical training, Learning transfer

## Abstract

**Background:**

During clinical practice, nursing students interact with healthcare professionals, clinical instructors, patients, and families, creating a complex experience that prepares them for their professional lives. This complexity may also lead to negative learning experiences; therefore, it is crucial to understand how students perceive the clinical environment. The purpose of this study was to determine nursing students’ experiences of transferring classroom learning into clinical practice.

**Methods:**

This descriptive phenomenological study was conducted with 40 s-year nursing students over a 14-week period during the 2019–2020 academic year using an online diary form. The inclusion criterion was taking the “Surgical Diseases Nursing” course for the first time. Data were analyzed using Colaizzi’s seven-stage method.

**Results:**

Students experienced both positive and negative emotional reactions during clinical practice. Practical skills were developed by observing healthcare professionals and integrating theoretical knowledge with clinical practice. The sense of belonging was reinforced when students applied their knowledge while participating in patient care. Structured around four major themes—emotionality, embodied practice, professional identity, and the clinical collective—the findings revealed that learning transfer is a transformative process of ‘embodied knowing,’ shaped by emotional intensity and interpersonal interactions. Nursing students’ participation, professional belonging, and motivation were increased by positive interactions with healthcare professionals, patients, and peers.

**Conclusions:**

The results explain how the gap between theoretical and clinical education can be addressed through the lens of experiential learning. To effectively reduce the theory–practice gap and transform clinical encounters into professional competence, nursing curricula must strategically incorporate structured mentorship, regular reflective practices, and robust emotional support systems. These findings provide a practical framework for nursing professors, clinical instructors, and healthcare professionals to create optimized, safer clinical learning environments.

**Supplementary Information:**

The online version contains supplementary material available at https://doi.org/10.1186/s12912-026-04990-5.

## Background

Clinical practice is essential in nursing education because it allows nursing students to apply their knowledge and develop various clinical skills. Learning outcomes in clinical practice are based on the theoretical education students receive in school and the practical skills they develop in skills laboratories [1]. 

The Bachelor of Science in Nursing (BSN) program consists of 4,600 h of classroom instruction and 2,300 h of clinical practice. In the clinical practicum, instructors provide theoretical guidance, while clinical nurses provide practical training in hospitals. In other words, instructors collaborate with clinical nurses to help nursing students achieve their learning goals. The educational support provided by clinical nurses to students occurs within the natural workflow of the clinical setting. In the present study, the nurse-to-student ratio was 1:5, while the instructor-to-student ratio was 1:40.

The clinical learning environment encompasses physical, psychosocial, and organizational components [2, 3]. Clinical learning plays a vital role in nursing education. In clinical practice, students interact with their environment physically, socially, and emotionally while working to achieve learning objectives. During this process, students are influenced by the physical conditions of the clinical environment and communicate with healthcare professionals, clinical instructors, patients, and families. Clinical education prepares students for professional nursing practice [2, 4].

During clinical education, students build confidence and a sense of belonging, develop organizational skills, and become part of a professional team [2, 3]. During clinical learning, both individual factors (such as motivation, concentration, and willpower) and extrinsic factors (such as the characteristics of the clinical environment, teaching methods, and teacher–student relationships) play an important role [5, 6]. The harmony between theoretical training and clinical practice is important for professional competence. Students who perceive significant differences between theoretical education and clinical practice are more likely to have negative learning experiences [4, 7, 8]. Therefore, there is a need to understand how students perceive clinical settings.

The transfer of learning from the classroom to clinical practice is a universal challenge in undergraduate nursing education, not only in Turkey but also across different educational systems. Studies from different countries consistently show that students struggle to integrate theoretical knowledge into clinical settings due to differences in role expectations, communication barriers, and clinical learning environments [9, 10]. These findings suggest that clinical learning is influenced by multiple factors, including professional identity formation, emotional safety, mentoring quality, and opportunities for supervised practice [9, 10]. This process is consistent with Kolb’s Experiential Learning Theory, which underlines that learning occurs through a strong and well-connected cycle of concrete experience, reflection, conceptualization, and active experimentation all of which are crucial mechanisms in clinical learning [11]. Building on this theoretical perspective, this study expands on the global literature by presenting evidence from Turkey, thus enabling comparison with international findings and contributing to a more comprehensive understanding of how undergraduate students perceive and experience clinical practice. Given the multidimensional nature of clinical learning, encompassing emotional, cognitive, interpersonal, and organizational elements, a qualitative phenomenological approach is well-suited to revealing the depth and complexity of students’ experiences. Understanding how students perceive clinical settings and how they convey information is crucial for improving curriculum design, strengthening clinical partnerships, and aligning educational strategies with real-world practice expectations.

This study presents a descriptive phenomenological investigation of nursing students’ experiences of transferring knowledge from the classroom to clinical practice. Accordingly, the study addressed the following research questions:

- What are the lived experiences of undergraduate nursing students in transferring classroom learning to surgical clinical practice.
- How do nursing students connect previous knowledge to new knowledge?

## Methods

### Study design

The study is grounded in Husserlian descriptive phenomenology, which seeks to describe the essence of lived experiences prior to any theoretical preconceptions or interpretations [12]. This approach was chosen because the aim of the study was to explore how students directly experience and make sense of their clinical experiences in their purest form. Husserlian phenomenology is uniquely suited for this inquiry as it emphasizes uncovering the universal structure of a phenomenon through the participants’ own statements. The design allows researchers to bracket their assumptions, focus on students’ lived experiences, and generate a rich description of the phenomenon of learning transfer. By utilizing this methodology, the research team aimed to capture the depth of the transition from classroom learning to clinical practice without imposing external professional biases.

### Setting and sample

The research was conducted at an undergraduate nursing school in Kahramanmaraş, where the first author works as a faculty member. Given the focus on lived experiences, convenience sampling was used to recruit participants. A trusting relationship between the participants and the researchers was considered beneficial for facilitating the expression of authentic lived experiences.

Total population sampling was used among eligible second-year students enrolled in the course. The study sample consisted of 40 students, representing the entire population of second-year students enrolled in the Surgical Nursing course during the study period. Thus, by capturing the entire eligible population, no students were excluded except those who declined participation; however, all students volunteered to participate. Since data collection relied on written logs rather than interviews, the sample size was considered appropriate and legally sufficient for phenomenological analysis to reach data saturation.

Data saturation was considered to have been achieved when no new meaning units or experiential descriptions emerged from the data. This point was reached after the review of approximately 34 clinical logs; however, all 40 logs were analyzed to confirm informational redundancy. The research team conducted consensus meetings to verify that saturation had been reached and that the corpus sufficiently represented variation in participants’ experiences.

The study was conducted during the 2019–2020 academic year, immediately before the COVID-19 pandemic. Clinical practice conditions during this period reflected standard in-person instruction without pandemic-related restrictions. This context enabled the researchers to capture students’ authentic clinical learning experiences.

Although the first author was a faculty member at the institution, several strategies were implemented to minimize power imbalances and ensure voluntary participation. First, data collection was conducted through an anonymous online data collection system, preventing researchers from linking any narrative to student identities. Second, the first author had no authority to evaluate clinical performance, as grading was conducted by a separate committee. Third, students were explicitly informed, both verbally and in writing, that participation was voluntary and that refusal would not affect their academic standing. Fourth, data analysis was conducted by the second author, who had no direct relationship with the students, providing an additional layer of objectivity.

### Ethical considerations

The research was conducted in accordance with the ethical principles of the World Medical Association’s Declaration of Helsinki [13]. Formal ethical approval was obtained from the Research Committee of the Faculty of Medicine of Kahramanmaras Sutcu Imam University prior to data collection. Institutional permission was also secured from the school administration. All students were fully briefed on the research purpose, and written informed consent was obtained from all participants on a voluntary basis. To maintain participant confidentiality, all clinical logs were securely archived in anonymized, password-protected cloud storage. The specific ethics approval protocol number and decision date are detailed in the Declarations section at the end of this manuscript.

### Participants/Measurements/Instruments

The sample consisted of 40 s-year (third-semester) nursing students enrolled in the compulsory 12 ECTS “Surgical Diseases Nursing” course during the 2019–2020 academic year. Prior to this course they had completed two practical courses: Basic Principles of Nursing (2 h of laboratory practice) and Fundamentals of Nursing (8 h of observational clinical practice). The inclusion criteria were (1) taking the “Surgical Diseases Nursing” course for the first time (2), volunteering to participate, and (3) keeping clinical logs during clinical practice in surgical units (8 h per week for a total of 14 weeks). During clinical practice, nursing students were expected to follow the principles of asepsis, wound care, and perioperative bleeding monitoring. As these interventions were practiced for the first time, the clinical experience represented a new learning context for the students. The first author was then the coordinator of the Surgical Nursing course. She introduced the study during the first theoretical session of the semester and invited students to participate. All students enrolled in the course volunteered to participate (*n* = 40).

### Data collection/Procedure

Data were collected using a clinical log system. Clinical logs are uniquely suitable for capturing deeply reflective learning experiences in real-time. Compared to traditional face-to-face interviews or focus groups, written clinical logs were preferred because they significantly reduce recall bias by allowing students to engage in immediate reflection right after their clinical exposure, while the experience is still fresh. Furthermore, this log-based approach provided a private space that eliminated peer pressure and social desirability bias often found in focus groups, ensuring more authentic self-disclosure. The researchers created an online platform where the clinical logs could be digitized and anonymized to eliminate the risk of recognizing students’ handwriting. This online diary form allowed students to narrate their clinical experiences freely and at their own pace. Each participant was provided with a random 8-digit code (ID) and a link to access a webpage containing an online form with the following open-ended questions: (1) What procedures have you observed today (2)? In which procedures have you participated today (3)? How did you feel in your clinical practice today (4)? Can you explain your experiences in general, both positive and negative (5)? What else would you like to say about today’s clinical practice?

The researchers asked all participants to answer these questions regularly after each day of clinical practice. However, they were also informed that they were free to write about any experiences related to their clinical practice without any word limit. When the clinical practice period ended, the web form was closed. Each log contained an average of 1,900 words, confirming that the written log method successfully generated rich, high-density qualitative data without the structural constraints of formal interview scheduling.

### Trustworthiness

The authors rigorously followed the quality criteria of credibility, transferability, dependability, confirmability, and reflexibility [14]. Credibility was achieved by (1) using a clinical log as a data collection method (2), giving all participants the flexibility to express their feelings whenever and as long as they needed, and (3) presenting direct quotations from the transcribed text. Transferability was ensured by describing the methodological design, data collection, and analysis processes in detail. Dependability was guaranteed by collecting data consistently over a 14-week period. In addition, a member-checking session was conducted with all participants at the end of the data analysis process to ensure that the findings accurately reflected their expressed experiences and interpretations. Confirmability was established as the research team collectively reviewed the findings (peer-check). They assured reflexibility by (1) receiving the clinical log anonymously and (2) getting the second author to analyze the data.

Although a formal external audit was not conducted, the authors maintained a clear audit trail by documenting coding decisions, analytic discussions, and theme development among the research team, which also supported bracketing and helped to minimize the risk that the authors’ own prior experiences might influence the analysis.

To operationalize Husserlian bracketing within this log-based design, the research team implemented a structured, reflective protocol to ensure that the analysis remained strictly faithful to the students’ written words. Before data analysis commenced, the authors held a pre-reflective session to explicitly identify and document their own prior assumptions, pedagogical expectations, and clinical experiences as nursing instructors. By making these implicit biases explicit, the researchers deliberately suspended (“bracketed”) them. Throughout the analysis, because there were no interview transcripts or non-verbal cues to interpret, the researchers bracketed their professional expectations by ensuring that every emergent code and theme was directly rooted in, and verified by, the literal text of the students’ logs. The first author refrained from initial coding, and the second author maintained a reflexive journal to continuously check that the interpretation did not over-extend beyond the participants’ actual written narratives, thereby safeguarding the descriptive purity of the phenomenological findings.

To further strengthen reflexivity and manage the potential power imbalances inherent in the researchers’ faculty roles, a strict boundary was maintained between pedagogical evaluation and data management. Although the first author was a faculty member at the institution, they had no direct grading responsibilities or evaluative authority over the participants during the study period. To safeguard the students from social desirability bias or fear of academic repercussions, the online data collection platform was managed entirely via randomized 8-digit identification codes, ensuring that the researchers received the clinical logs in a fully anonymized format. Furthermore, the second author, who held no teaching or institutional ties to the participants, conducted the primary data analysis independently. The research team engaged in continuous peer-debriefing sessions to challenge each other’s interpretations, ensuring that the final themes were anchored exclusively in the students’ authentic written voices rather than the authors’ institutional expectations.

### Data analysis

The second author was responsible for conducting the data analysis. She has formal training in qualitative research methods, including phenomenology, and extensive experience in qualitative data interpretation. Her role as a researcher with no teaching or evaluative responsibilities in the program further minimized potential bias during the analytic process. Nevertheless, all authors contributed to different phases of the analysis.

The data were analyzed using Colaizzi’s seven-step phenomenological method [12]. First, the first two authors read each log repeatedly to gain a general understanding of the participants’ lived experiences. Second, they extracted significant statements related to the phenomenon under investigation. Third, meanings were formulated from these significant statements. Fourth, the formulated meanings were organized into clusters of themes. Fifth, the themes were integrated into an exhaustive description of the phenomenon. Sixth, the fundamental structure of the phenomenon was formulated. Seventh, the findings were returned to the participants to obtain feedback and validate the results.

Qualitative data were managed and analyzed using MAXQDA 2018. To ensure a rigorous and transparent analysis, the transition from raw text to final themes followed a strict multi-stage coding process. In the first open coding phase (Steps 2 and 3 of Colaizzi’s method), numerous significant meaning units were systematically identified from the raw text, resulting in an initial pool of 46 open codes. Due to the semi-structured nature of the dataset, excessive fragmentation was avoided during this initial step while maintaining analytical sensitivity. In the second phase of analysis (Step 4 of Colaizzi’s method), these initial 46 meaning units were subjected to an analytical reduction and constant comparison process. Codes with conceptual overlaps were combined, and duplicate categories were merged to avoid redundancy. This refinement and consolidation process successfully reduced the initial pool into 21 finalized conceptual codes. Through an iterative process of coding, comparison, and categorization of these 21 refined codes, the research team developed a thematic structure consisting of four main themes and 13 sub-themes. To enhance methodological rigor and ensure analytical triangulation, all authors participated in repeated meetings throughout the analytic process to compare coding decisions, discuss emerging categories, refine thematic clusters, resolve discrepancies through consensus, and confirm the final thematic structure.

To ensure methodological rigor and strict compliance with international reporting standards, the study design, research team characteristics, and data analysis procedures were fully aligned with the Consolidated Criteria for Reporting Qualitative Research (COREQ) guidelines [15]. A detailed mapping of Colaizzi’s seven-step phenomenological method against the specific COREQ items is provided in Supplementary Table S1.

## Results

Learning transfer for students is not merely the relocation of theoretical knowledge into the clinical field; it is a holistic transformative process in which theoretical knowledge evolves into ‘lived and embodied knowledge’ through the emotional intensity of the clinical environment, escalating responsibilities, and interpersonal interactions. In this process, the student transcends the role of a passive observer to become a nurse who embodies a professional identity and assumes the ethical responsibility of care.

The findings answered the research questions as follows: (1) Nursing students’ experiences in surgical clinics encompassed emotional, social, and cognitive components, which were reflected in four interpretive themes: The Lived Emotionality of Clinical Encounters, From Theoretical Knowing to Embodied Practice, The Becoming: Shaping Professional Identity, and Being Part of the Clinical Collective. (2) These experiences influenced learning transfer by reinforcing confidence, bridging the gap between theory and practice, and facilitating the formation of professional identity. (3) Students connected previous knowledge to new clinical learning through observation, guided practice, reflective interpretation, and the integration of theoretical concepts into clinical decision-making processes.

### Demographic data

Participants had a mean age of 20.90 ± 1.03 years. Most participants were female (*n* = 28). Table 1 shows the demographic characteristics of the participants.

**Table 1:** Demographic characteristics of the participants (*N* = 40)

Demographic characteristic	Categories	*n* (%) / Mean ± SD
Gender	Female Male	28 (70.0%) 12 (30.0%)
Age (Range 20–23)		40 (100.0%) /20.90 ± 1.03
Academic Year	Second Year	40 (100.0%)
Course Enrollment	Surgical Nursing	40 (100.0%)

#### Themes

The essential structure of the phenomenon revealed that learning transfer during clinical practice was experienced as a transformative process in which nursing students moved from theoretical understanding to embodied clinical knowledge. This process was shaped by emotional vulnerability, increasing responsibility, and interpersonal interactions within the clinical environment, gradually contributing to the development of professional identity and confidence.

The analysis revealed four main themes and 13 sub-themes. The main themes were: (1) The Lived Emotionality of Clinical Encounters (2), From Theoretical Knowing to Embodied Practice (3), The Becoming: Shaping Professional Identity, and (4) Being Part of the Clinical Collective. This section presents the main themes and sub-themes along with their interpretive descriptions. Table 2 shows the main themes, sub-themes, and representative quotations.

**Table 2:** Main themes, sub-themes, and quotations

Main themes	Sub-themes	Quotations
1.The Lived Emotionality of Clinical Encounter	*Reactions to Death* *Feeling Worried*	“I had a patient on his deathbed. I mean, his case was completely hopeless. He had pancreatic cancer… I ended up at his door at least 30 times throughout the day. I wasn’t actually sorry he was dying. I was sorry that I had to watch him die.” (P-21) “The doctor delivered the baby after some effort. But the baby’s pulse had stopped. I felt horrible at that moment. The doctors took him and intervened immediately. They put an oxygen mask or something on him. They gave him a heart massage. Of course, I was praying all the time. God, please let him live. My eyes were full of tears, and I wanted to cry. (P-18) “I asked the nurses about the patients who were in the clinic last week and not in the clinic today. I wish I hadn’t…the patient whose story I found so interesting had died. Very painful… I don’t know, maybe it was because I was experiencing new things in my life but today was very painful. It was a day when my words got stuck in my throat” (P-11). “When I collect blood samples or do invasive procedures, I worry that I might hurt the patient.” (P-4) “In our first internship, I was worried when a friend in the operating room fainted. I was afraid of experiencing the same thing.” (P-16) “This will really be a different experience for me. I have always wanted to watch an operation. Of course, I was also nervous about what and how I would do it. I was afraid of making a mistake.” (P-18)
	*Feeling Brave* *Feeling Happy*	“A patient needed an intravenous line. The nurses told me that I could do it on my own. I felt confident, and I got to do it. I felt thrilled. I felt brave.” (P-28) “A patient thanked me and even told me that he knew I was a student and that I was so excited that he let me start an IV. He said, otherwise, he was terrified of needles. I cannot explain how happy I was when he said those things.” (P-1) “A patient of mine was getting discharged. On her way out, she thanked me. The interesting thing is that when she left the clinic, she asked my permission and thanked me. I liked that very much.” (P-2)
2.From Theoretical Knowing to Embodied Practice	*Learning by Observing Healthcare Personnel*	“First, the sterile nurse washed her hands and entered the room. She opened her bundle. She put on her gown and gloves in a sterile way. Then she put her stuff on the table. She was cautious about hygiene. At school, we’d learned about the duties of a sterile nurse. Observing her made me think that I knew and learned something. I felt the desire to learn more and put it into practice.” (P-30)
	*Integrating Theory with Clinical Practice*	“Before entering the operating room, surgical team members washed their hands according to the surgical hand-washing technique. We’d learned and practiced that technique at school. Watching them wash their hands in that way helped me learn it permanently. If I had not observed health care professionals here, maybe I would have forgotten what I’d learned at school; I don’t know.” (P-14) “I saw many instruments in the operating room and learned what they were for. I learned how to wear the gown and how to put on gloves. I understood very well that sterilization is very important and how it should be done. I examined the ventilation system and checked the temperature in the operating room. If it was too cold, I found out why. We’d learned these things in the surgery course, but here we learned them thoroughly. I will follow the lessons more carefully from now on.” (P-25)
	*Developing Practical Skills*	“After the patient was moved to the bed, I drew up an observation form to be followed up every 1 hour and 15 minutes. I had the first follow-up and noted it down. Me and the nurse prepared liquids and applied what needed to go in order. I checked whether the patient had post-op pain. They had an ice battery. I explained how to use it. I mean, me and the nurse gave the patient training.” (P-3) “We know how to use the electrocardiography (ECG) machine because there was always a patient who needed an ECG in almost every department we visited.” (P-22).
3.The Becoming: Shaping Professional Identity	*A Sense of Belonging to the Nursing Profession*	“I selected a patient for my care plan in the orthopedic ward. While reviewing the patient’s file, I noticed that all interventions we’d learned about before, during, and after surgery were all listed there. I thoroughly examined which ones had been applied to the patient, and they were no different from what we’d learned. At that moment, I felt proud of what I’d learned. I was learning and getting closer to becoming a nurse step by step.” (P-36) “I was in the operating room today. I was told that I could watch an operation. Before entering the surgery room, I was asked to put on a sterile gown. Remembering what I’d learned at school and what I observed in the operating room, I put on the sterile gown with the help of the nurse there. I felt very different; I felt like I belonged to that team. I think I will become an operating room nurse.” (P-2) “I noticed that the patient’s oxygen saturation level had decreased, and so I checked it. The oxygen mask was not properly fitted. I adjusted the mask properly, and after that, the oxygen level returned to normal. While I was receiving training on oxygen applications at school, my professor talked about it, and I immediately remembered what he’d taught us. Everything that is taught in school is very important. We are not just providing care or treatment to people. We touch their lives, and we learn many lessons from their lives. I’ve fallen in love with my profession once again.” (P-21)
	*Adherence to Ethical Principles*	The most interesting thing that happened today was that a staff member’s father came to our department because he was sick. The nurses took special care because he was the father of a staff. This was upsetting. After all, everyone is equal; it was not right for them to discriminate.” (P-15) “I obtained permission from patients before collecting blood samples or inserting an IV line.” (P-17)
4.Being Part of the Clinical Collective	*Interaction with Faculty Members*	“Today, besides these procedures, we went to the resuscitation room with our internship supervisor and examined the devices and instruments there. Our supervisor informed us about resuscitation. Then she asked some questions, and I answered them or made deductions based on logic. Today was more productive.” (S 14) “I was inserting an IV into a patient when my professor came up to me. He asked me some questions, but I couldn’t answer them correctly, so he told me what to do. He gave me some information and then told me what I should research. He said that I should evaluate every piece of information about the patient. I should act like a detective, curious and observant. I liked that.” (P-9)
	*Interaction Within the Team (Nurses)*	“I started filling out the forms of a patient who was going to have surgery. A nurse helped me with it and explained the parts we didn’t know about. The nurses were so positive that it affected us positively. We enjoyed caring for all of our patients.” (P-35) “The nurses guided us. They then assigned a nurse to each student. They taught us how to collect blood samples and told us to watch them carefully. I watched my own nurse for a while to see how she collected blood samples, then our guide nurse asked me to collect a blood sample from a patient. I was excited but didn’t show it to the patient. The nurse corrected my mistakes by telling me that I pressed the vacutainer a little too hard. My nurse’s support gave me confidence.” (P-35)
	*Communication with Patients*	“Half an hour after we returned to the operating room, there was an abortion operation. They brought the female patient in, and we were again in front of the door. The woman looked at us with tears and said, ‘Please pray for me, will you?’ I couldn’t look at her face at that moment because she said it so sincerely, and it was obvious she was in pain.” (P-1) When I see elderly patients and patients in pain, I can’t help but think about my family.” (P-3) “It is awful to be sick and feel discomfort, so it is really important to understand the condition of patients and to treat and care for them by empathizing with them. That’s what I’ve learned today.” (P-23)
	*Peer-to-Peer Communication*	“There was a newcomer at our clinic. I tried to help her get used to the clinic, the nurses, and the patients.” (P-2) “This is the third week of my internship. This week, we’ve been assigned to new posts. I’ve been assigned to the orthopedics unit. I was new there, but my two friends had been there for three weeks already. They both helped me a lot because I was new there. So, it was easy for me to get used to the unit The presence of my friends made me feel very comfortable. I felt like I belonged to the clinic. It was nice to feel the support of my friends.” (P-8)

1. The lived emotionality of clinical encounters

Participants expressed positive or negative emotions in their clinical logs. Adverse emotional reactions were related to “*Reactions to Death”* and “*Feeling Worried*” and positive emotional reactions were related to “*Feeling Brave*” and “*Feeling Happy*.”

2) From theoretical knowing to embodied practice

Participants stated that they attained numerous individual learning outcomes during clinical practice, such as *Learning by Observing Health Personnel*, *Integrating Theory and Clinical Practice*, and *Developing Practical Skills.*

Under the “*Learning by Observing Health Personnel*” sub-theme, participants noted that they developed clinical skills by observing healthcare professionals.

Under the sub-theme “*Integrating Theory and Clinical Practice*,” participants remarked that they attained more clinical learning outcomes and became more motivated as they watched other healthcare professionals in action and put what they learned in theory in school into practice in clinical settings.

Under the sub-theme “*Developing Practical Skills*,” participants noted that they developed numerous clinical skills and became more confident because they kept practicing them again and again during clinical practice.

3) The becoming: shaping professional identity

The main theme “*The Becoming: Shaping Professional Identity*” consisted of two sub-themes: “*A Sense of Belonging to The Nursing Profession*” and “*Adherence to Ethical Principles.*”

Under the sub-theme “*A Sense of Belonging to The Nursing Profession*,” participants stated that clinical practice, that is, putting theory into practice in clinics and caring for patients, helped them develop a sense of belonging to the nursing profession.

Under the sub-theme “*Adherence to Ethical Principles*,” participants expressed that they were disturbed by the unethical behavior of some healthcare professionals and added that they worked hard to stick to ethical principles during clinical practice.

4) Being part of the clinical collective

The main theme “*Being Part of The Clinical Collective*” consisted of four sub-themes: *Interaction with Faculty Members*,* Interaction Within the Team (Nurses)*,* Communication with Patients*, and *Peer-to-Peer Communication*.

Under the sub-theme “*Interaction with Faculty Members*,” participants remarked that the fact that instructors visited them during clinical practice contributed significantly to their learning.

Under the sub-theme “*Interaction with Nurses*,” participants noted they were happy working with nurses and being involved in their routine interventions. They also added that nurses helped them recognize and learn from their mistakes.

Under the sub-theme “*Interaction with Patients*,” participants noted that interacting with patients helped them empathize with the individuals under their care and make meaningful inferences from those encounters.

Under the sub-theme “*Peer-to-Peer Communication*,” participants remarked that they lent support to each other and welcomed newcomers, which helped them get used to the setting quickly.

## Discussion

The findings of this study provide a deep understanding of the learning transfer experiences of undergraduate nursing students in surgical units, framed as a holistic, transformative journey. The results illustrate that learning transfer is not merely the mechanical relocation of theoretical knowledge into the clinical field; rather, it is a dynamic process where static concepts evolve into ‘lived and embodied clinical wisdom’ through emotional intensity, escalating responsibilities, and interpersonal interactions. This transformative trajectory aligns significantly with Kolb’s Experiential Learning Cycle, providing a structural map of how students bridge the gap between classroom theory and real-world practice through a continuous loop of reflection and action.

### The lived emotionality of clinical encounters

Participants reported experiencing anxiety, stress, and sadness when witnessing death or providing care to terminally ill patients. Previous studies have similarly shown that nursing students involved in end-of-life care may experience anxiety, fear, stress, and low self-efficacy [16–19]. Some studies have indicated that nursing students feel anxious and overwhelmed about end-of-life care despite having basic knowledge of patient care [20, 21]. Taylor, Dihle, Hofsø, and Steindal (2020) reported that both undergraduate and graduate students described “encounters with death” as bad, very bad, and an experience they would never forget [22]. Zheng, Yang, Zhou, and Zhu (2023) found that new nurses were also uncomfortable and angry about end-of-life care and felt inadequate when communicating with patients’ family members [23]. Our results suggest that caring for terminally ill patients causes nursing students to feel stress, sadness, anxiety, and fear, as end-of-life care makes them feel helpless rather than enabling them to perceive it as an opportunity for learning or professional duty. Therefore, end-of-life care is a unique experience in which nursing students feel hopeless. From the perspective of Kolb’s Experiential Learning Theory, these encounters with death create profound “Concrete Experiences” that challenge students’ existing knowledge. The emotional intensity of these moments triggers a shift to “Reflective Observation,” where students use their clinical records to process these lived experiences. By reflecting on these challenging encounters, students can begin to bridge the gap between theoretical ethics and the ‘concrete’ reality of end-of-life care [11]. To support this transition, providing compassion and self-compassion training can be a crucial pedagogical strategy. Such training enables students to manage the emotional burden of their ‘Reflective Observation’ more effectively, ultimately facilitating the successful transfer of ethical knowledge into compassionate clinical practice [5].

Participants reported feeling worried due to perceived gaps in their professional knowledge and manual skills, particularly regarding patient safety and the risk of making errors. However, this concern for patient safety may also encourage nursing students to act more cautiously when performing delicate interventions. Participants also experienced worry when witnessing peers in adverse situations, suggesting that they experienced state anxiety [24]. Nursing students often experience state anxiety due to high competency expectations from professors, nurses, and patients. However, when provided with one-to-one guidance and clear role expectations, this anxiety can transform into increased confidence and improved performance [25]. Within Kolb’s theory, this anxiety arises during the ‘Concrete Experience’ stage, where the surgical unit’s unpredictability tests student readiness. When managed through guidance, this anxiety becomes a precursor to ‘Active Experimentation,’ as the cautiousness born of worry ensures that initial independent clinical attempts are performed with heightened cognitive focus [11].

Extrinsic environmental factors, such as patients’ good wishes and nurses’ encouraging remarks, motivate students to learn and engage in clinical processes. Successful performance of clinical interventions further boosts nursing students’ confidence. Ultimately, these positive interactions foster active participation in clinical learning and ensure successful procedural outcomes [6, 26, 27]. Yua and Jia (2023) argue that nursing students generally fear negative evaluations, although some may also experience anxiety related to positive evaluations [28]. Our findings suggest that encouraging remarks positively influence nursing students, increasing their willingness to participate in clinical learning. These positive emotional states facilitate the ‘Active Experimentation’ phase of Kolb’s cycle. When students feel brave and supported, they are more likely to apply theoretical knowledge in real-world scenarios, thereby reinforcing their professional identity and transforming abstract concepts into successful clinical actions.

### From theoretical knowing to embodied practice

The sub-themes involving observation, theory integration, and skill development indicate that students gain greater self-confidence and professional belonging when allowed to perform clinical interventions independently. Participants reported more lasting learning when observing nurses apply classroom knowledge in real settings. This suggests a minimized theory-practice gap, which directly enhances students’ professional skill development [4, 8]. Observation is a purposeful, meaningful, and active process that requires concentration. However, nursing students must also apply their cognitive skills in clinical settings [29, 30]. By observing healthcare professionals, nursing students successfully connect classroom learning with real clinical practice. Ying et al. (2023) argue that student engagement is significantly higher in clinical settings than in traditional classroom learning [6]. Similarly, our findings suggest a strong, reciprocal relationship between clinical and classroom learning, where active observation makes learning more meaningful and enhances overall academic engagement. In light of Kolb’s model, these outcomes illustrate the transition from ‘Reflective Observation’ to ‘Abstract Conceptualization’ (signifying Objective Conceptualization). Through active observation in the surgical unit, students critically analyze the alignment between the nurse’s actions and classroom principles. This cognitive processing allows them to systematically detach from subjective emotional responses, enabling the formation of objective concepts that transform static theory into a dynamic, integrated part of their clinical reasoning.

### The becoming: shaping professional identity

Participants reported a stronger sense of belonging to the nursing profession when healthcare professionals entrusted them with greater responsibility, reinforcing how clinical learning consolidates classroom knowledge. Such commitment remains essential for overall professional success and continuity [31–33]. Our findings suggest that when nursing students independently practice and develop clinical skills, they experience a stronger sense of professional belonging. Consistent with this, previous research shows that applying theory in practice directly enhances students’ professional satisfaction [1]. Similarly, translating theoretical knowledge into clinical practice yielded greater professional satisfaction and belonging. Regarding the sub-theme “Adherence to Ethical Principles,” participants prioritised patient privacy, informed consent, and equal treatment, suggesting that academic education effectively supports students in upholding patients’ rights. Viewed through Kolb’s theory, this professional growth marks the transition into ‘Active Experimentation,’ where students test their ‘Abstract Conceptualization’ (signifying Objective Conceptualization) of ethical standards in real-world care. By independently operationalising principles like informed consent and privacy, students do not merely perform tasks; they embody the nurse’s role. This successful experimentation with their professional persona reinforces their sense of belonging, moving them from ‘knowing’ about nursing to ‘becoming’ a nurse within the clinical collective [11].

### Being part of the clinical collective

Research indicates that effective communication with instructors enhances the benefits students derive from clinical education [34, 35]. Similarly, participants reported making greater efforts to learn and perform duties due to positive relationships and consistent support from instructors and nurses, which also enhanced their enthusiasm for clinical practice. This willingness to exert effort remains a core component of professional commitment [33]. Nursing students may feel nervous when questioned by instructors; however, learning is facilitated when accompanied by guidance and explanations. Instructors play a pivotal role in addressing questions and providing constructive feedback. Conversely, students can feel demotivated to learn when unable to answer or when receiving inadequate feedback [9, 36–38]. Consequently, participants reported enjoying the learning environment more when instructors clarified unanswered questions. Within Kolb’s learning cycle, these interpersonal interactions function as the primary support system for ‘Reflective Observation’ and the transition to ‘Active Experimentation.’ Positive feedback and guidance from educators act as a protective bridge, allowing students to reflect on ‘Concrete Experiences’ without the paralyzing effect of evaluation anxiety. When clinical nurses and faculty members provide a supportive environment, they facilitate the students’ ability to test conceptual knowledge safely, ensuring the learning cycle continues toward mastery rather than being interrupted by demotivation [11].

Regarding the “Peer-to-Peer Communication” sub-theme, participants reported sharing clinical experiences with one another, noting that support from more experienced peers motivated them to learn and complete assigned tasks. Previous research similarly demonstrates that peer support positively influences the learning process [25, 39]. Therefore, healthcare professionals should encourage peer support within clinical environments to help nursing students acquire skills and perform assigned duties. Theoretically, peer-to-peer communication provides a vital collaborative space for ‘Reflective Observation’ within Kolb’s cycle. By sharing lived experiences, students engage in collective reflection that helps externalize ‘Concrete Experiences’ and validate findings before moving toward ‘Abstract Conceptualization’. This shared reflection reduces the clinical setting’s emotional burden while accelerating the transformation of individual observations into common professional knowledge [11].

**Fig. 1:**
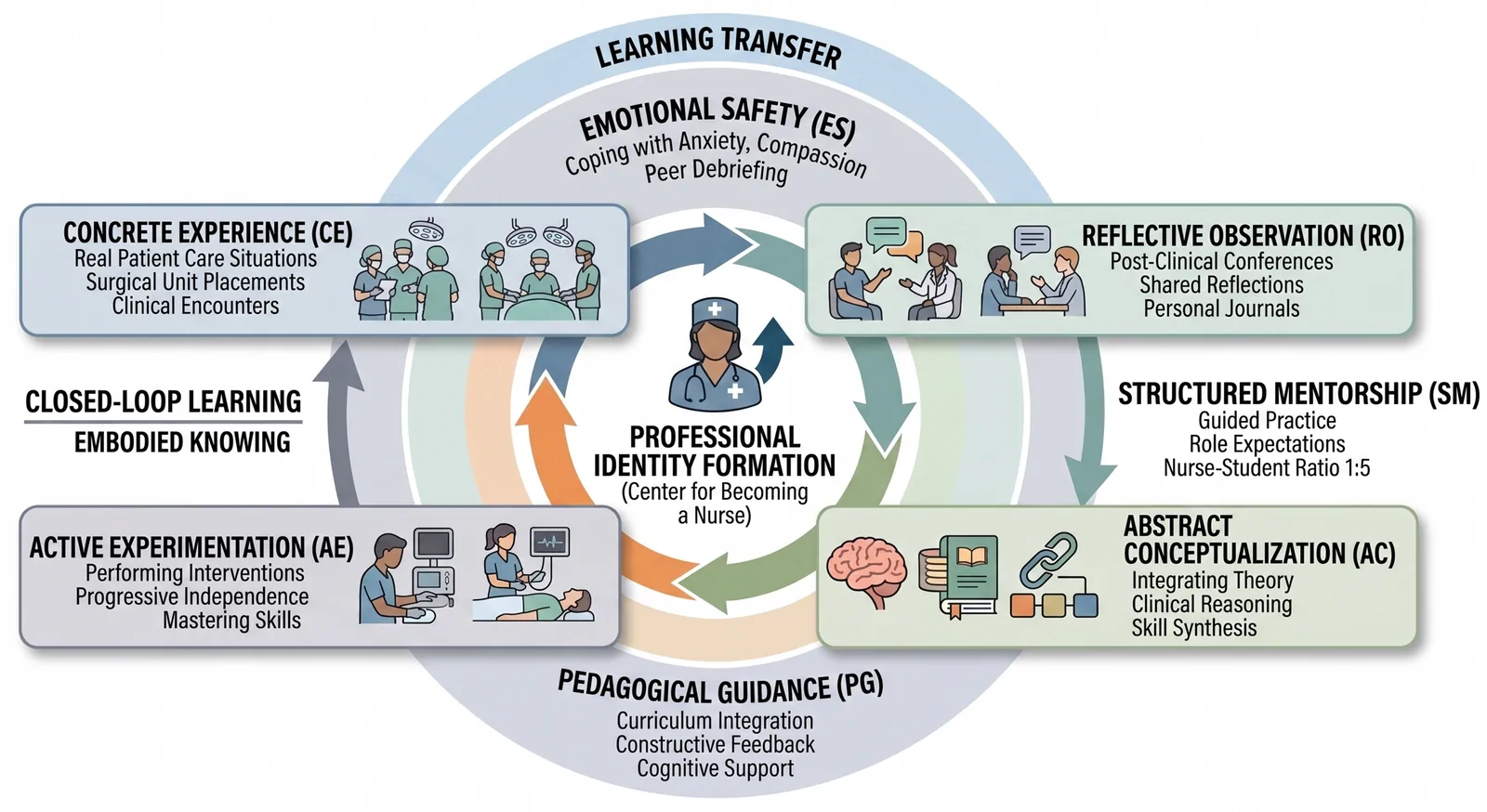
The experiential transformation model of learning transfer in surgical nursing education: integrating Kolb’s experiential learning cycle with professional identity formation and supportive pedagogical frameworks

Our findings showed that empathy plays a vital role in patient-student relationships. Consistent with previous literature, emotional interaction with patients can increase nursing students’ learning motivation and support clinical skill development [9]. Our participants expressed a desire to communicate with patients but lacked the practical skills to do so, which acted as a distinct barrier to learning transfer. Similarly, Rosliany et al. (2023) highlighted that nursing students require structured support and guidance when communicating with patients [40]. These findings suggest that nursing students should be taught effective communication skills to provide appropriate care and meet patients’ needs. Within Kolb’s framework, these empathetic encounters represent ‘Concrete Experiences’ with high emotional valence. The communication difficulties students face, despite their desire to empathize, trigger a crucial ‘Reflective Observation’ phase where they confront clinical limitations. Addressing this barrier through structured training allows students to transform raw emotional experiences into ‘Abstract Conceptualization,’ facilitating a more sophisticated and compassionate professional identity [11] (Fig. 1).

Overall, the findings clearly address the three research questions. First, the study identified the types of experiences nursing students encounter in surgical clinics, including emotional reactions, interpersonal interactions, and professional learning processes. Second, the findings demonstrate how these experiences support or challenge the transfer of learning from the classroom to clinical practice. Finally, the findings illustrate how students connect prior theoretical knowledge with new clinical knowledge through observation, guided practice, and repeated exposure to real patient care. These findings collectively suggest that learning transfer is a non-linear, experiential journey where theoretical concepts are transformed into ‘embodied’ clinical wisdom through the continuous loop of Kolb’s cycle.

Interactive and participatory classroom education is critical for effective clinical learning. Healthcare professionals should support nursing students and encourage their active engagement in clinical practice. They should also provide clear explanations of procedures and interventions. Such support helps students develop positive relationships with healthcare professionals and instructors and increases their enthusiasm for acquiring new knowledge and skills. In addition, both theoretical and clinical nursing educators should encourage nursing students to take an active role in their own learning. From a pedagogical standpoint, clinical training should be structured to allow students adequate time for ‘Reflective Observation’ after complex encounters, such as end-of-life care or surgical procedures, to ensure that ‘Concrete Experiences’ are effectively translated into professional competence. Finally, the nurse–student ratio in clinical settings should ideally not exceed 1:5 to ensure effective observation and feedback. Adhering to this ratio is essential for maintaining the quality of mentorship required to guide students through the transition from observation to independent ‘Active Experimentation. To operationalize these findings into nursing education, a structured pedagogical approach is required. The specific strategies for emotional support, mentorship, and reflective practices are synthesized in the Implications Framework for Nursing Curriculum, presented in Table 3.

**Table 3:** Implications framework for nursing curriculum

Dimension	Proposed Strategy	Expected Outcome
Emotional Support	Integration of self-compassion and emotional intelligence modules into the curriculum for high-stress situations such as end-of-life care and fear of clinical error.	Transformation of anxiety and helplessness experienced during the ‘Concrete Experience’ stage into motivational drivers rather than barriers to learning transfer
Structured Mentorship	Maintaining a fixed nurse-to-student ratio of 1:5 and providing “pedagogical guidance” training for clinical preceptors	Ensuring a safe environment for ‘Active Experimentation’ where professional identity is reinforced through ‘embodied knowing’ under expert guidance
Pedagogical Tools	Mandatory use of reflective journals based on Kolb’s learning cycle, supported by structured and constructive feedback sessions.	Facilitating the transition from ‘Reflective Observation’ to ‘Abstract Conceptualization’ (signifying Objective Conceptualization), allowing students to systematically detach from subjective emotional responses and synthesize clinical observations into logical, lasting clinical reasoning
Curriculum Design	Aligning theoretical surgical nursing courses with clinical rotations or supporting them through high-fidelity “case-based simulations.”	Minimizing the ‘theory-practice gap’ and accelerating the transfer of classroom knowledge into real-world clinical performance

### Limitations

There are several limitations to consider when interpreting our findings. First, data were collected via written clinical records rather than face-to-face interviews, limiting the examination of nonverbal cues, follow-up questions, and deeper contextual depth. Second, despite mitigation strategies like anonymization and independent analysis, the first author’s role as an instructor may have influenced student participation or narrative content. Third, reliance on a single data source precluded data triangulation, potentially reducing interpretive richness. Fourth, this study was conducted in a single-institutional context within a single nursing school; thus, the findings reflect specific programmatic dynamics and may not be transferable to other nursing programs without contextual comparison. Finally, data collection occurred immediately before the COVID-19 pandemic under routine conditions. Post-pandemic changes in clinical access, emotional stress, and training models might yield different outcomes, suggesting that future research should compare pre-, intra-, and post-pandemic cohorts to capture these evolving dynamics.

## Conclusion

Clinical practice bridges the gap between classroom theory and real-world application, significantly reinforcing nursing students’ academic integration. Key outcomes indicate that positive relationships within the clinical collective directly foster a stronger sense of professional belonging, while emotional stressors are effectively mitigated by supportive nursing staff. In terms of practical implications, these findings suggest that nursing institutions must move beyond traditional supervision by strategically embedding structured mentorship and regular reflective practices into the curriculum to foster a safer learning environment. To maximize learning transfer and cultivate a resilient professional identity, future research should utilize longitudinal designs to track the long-term sustainability of identity formation and conduct multi-center comparative studies across diverse institutional contexts.

## Supplementary Information

Below is the link to the electronic supplementary material.

Supplementary Material 1

## Data Availability

Due to ethical considerations and the need to protect participant confidentiality, the qualitative data generated during this study are not publicly available. However, they may be obtained from the corresponding author upon reasonable request.

## Abbreviations

BSN: Bachelor of Science in Nursing
COREQ: Consolidated Criteria for Reporting Qualitative Research
COVID-19: Coronavirus Disease 2019
ECG: Electrocardiography
ECTS: European Credit Transfer and Accumulation System
ID: Identification code
