# Supplementary material for: Learning transfer from classroom to clinical practice in surgical units: a descriptive phenomenological study of undergraduate nursing students
Source: BMC Nurs. 2026 Jul 7;25:902. doi: 10.1186/s12912-026-04990-5 (PMC13625415; doi:10.1186/s12912-026-04990-5)
Supplement: Supplementary file 1 [file 12912_2026_4990_MOESM1_ESM.docx]

**Clinical Log Guide:** Online form with the following open-ended questions

1) What procedures have you observed today?

2) In which procedures have you participated today?

3) How did you feel in your clinical practice today?

4) Can you explain your experiences in general, both positive and negative?

5) What else would you like to say about today's clinical practice?

6) You are free to write whatever you felt regarding your clinical practice without any word limit.
